# Verbal Descriptions of Cue Direction Affect Object Desirability

**DOI:** 10.3389/fpsyg.2019.00471

**Published:** 2019-03-11

**Authors:** Jason Tipples, Mike Dodd, Jordan Grubaugh, Alan Kingstone

**Affiliations:** ^1^School of Social Sciences, Psychology, Leeds Beckett University, Leeds, United Kingdom; ^2^Department of Psychology, University of Nebraska – Lincoln, Lincoln, NE, United States; ^3^National Institutes of Health, Bethesda, MD, United States; ^4^Department of Psychology, University of British Columbia, Vancouver, BC, Canada

**Keywords:** gaze, arrows, liking, attention, cue

## Abstract

Approach-avoidance behaviors are observed across a broad range of species. For humans, we tend move toward things we like, and away from things we dislike. Previous research tested whether repeatedly shifting visuo-spatial attention toward an object in response to eye gaze cues can increase liking for that object. Here, we tested whether a gaze-liking effect can occur for verbal descriptions of looking behavior without shifts of attention. Also, we tested the gaze specificity hypothesis – that the liking effect is specific to gaze cues – by comparing the effect of different types of cue (pointing gestures and arrow cues). In Experiment 1, participants (*N* = 205) were split into 5 groups according to the type of cue that was described as directed either toward or away from an object. The results show that (1) attention is not necessary; the liking effect was recorded for verbal descriptions of looking, (2) the effect also occurs for descriptions of pointing and arrows, and (3) the liking effect is enhanced for gaze cues compared to arrows, consistent with the gaze specificity hypothesis. Results from a further experiment suggest that the effect is not due to demand compliance. We conclude that the gaze-liking effect occurs for verbal descriptions of eye gaze. Indeed, because our method bypasses altogether the use of visual cues, objects, and shifts in visual selective attention, our paradigm appears to be more sensitive at tapping into the fundamental approach-avoidance response that mediate the implicit liking effect. As such, it offers new opportunities for research investigations in the future.

## Introduction

Approach-avoidance reactions are a fundamental aspect of human behavior – we move away from things we dislike and toward things we like. Because people have the capacity to infer mental states from other people, it follows that that people might use other people’s approach-avoidance behavior as a basis for their own decisions and preferences. If I see you move toward an object, then that may indicate that I will also like that object. Similarly, if I see your eyes gaze toward an object then I might infer that the object is desirable.

To study the effects of gaze direction on object liking one innovative study (Bayliss et al., 2006) used an implicit learning task, in which shifts of attention were expected to increase object desirability. During the task, participants were asked to classify an object that appeared to the left or right of a face. The objects were presented in different colors. Across trials, the face looked consistently either toward or away from one of the colored versions of the object. In a final block of trials, participants both classified the object and rated the extent to which they liked the object. The key finding was increased liking for gazed-at objects – people rated objects as more likeable if they saw people gaze repeatedly toward rather than away from the objects.

This effect appeared to reflect an implicit process because participants did not report being aware that gaze direction had been deliberately manipulated to look toward certain objects, and away from other objects. Support for the idea that people had shifted attention in response to the gaze cues came from analyses of reaction times – reaction times were faster when the eyes appeared to look toward the location of a target (congruent trials) compared to away from the target (incongruent trials). Finally, and in keeping with the idea of a specialized social perception process, the effect of cue direction on liking did not occur when the gaze cues were replaced by arrows (Bayliss et al; Experiment 2). Other studies have extended this work by, for example, varying attributes of the face that are relevant to social interaction including expression (Bayliss et al., 2007), trustworthiness (King et al., 2011; Treinen et al., 2012) and attractiveness (Strick et al., 2008).

Despite the intuitive plausibility of the gaze-liking effect, a recent replication attempt (Tipples and Pecchinenda, 2018) of the gaze-liking effect indicated that the effect is much smaller (*d*_z_ = 0.02) than originally thought (d_z_ = 0.94). One possible reason for the small effect size is that the implicit learning task used in previous research does not directly assess the question of interest “Do participants prefer objects that are looked at by other people?” Instead, the task requires (implicit) learning of a relationship between gaze direction and objects, via shifts in attention. Some participants may fail to learn the gaze-object relationship – perhaps because they do not shift and maintain their attention to the object on some trials – and consequently, liking does not increase for gazed-at objects.

Our hypothesis for the current research is that the method used to increase liking – learning the gaze-object relationship via shifts in attention – is not necessary for gaze and other cues to affect liking for objects. Instead, all that is required is to present cue information and ask for ratings of likeability. We tested this idea across 5 groups of participants in which we varied the type of cue information. To test for a relation between cue direction and object liking we used verbal descriptions of gaze and other types of cue information. Our reason for using verbal descriptions was that mental states can be described by verbal communication (for a review see; Saxe, 2006) and language is related the development of mental state attribution (Dunn and Brophy, 2005). Our second hypothesis relates to the type of cue used to direct attention. Following previous research, we tested the gaze specificity hypothesis; that the gaze liking effect is unique to eye gaze cues. Specifically, cue type was varied between participants by allocating participants to one of five different groups. These are described below in detail.

Participants in the first group (LOOK – IMAGE) were asked to rate images of household objects that were paired with verbal descriptions of actors gazing toward (“Michael looked toward”) or away from (“Michael looked away from”) an image of an object. For the second group, (LOOK – WORD), the actors were described gazing toward or away from a verbal description of the object (e.g., “a screwdriver”) rather than a picture of the object. For the third group (POINT) the word “pointed” replaced the word “looked” (“Michael pointed away from”). A fourth group (ARROW) received verbal descriptions of an arrow pointing toward (“An arrow pointed toward”) or away from (“An arrow pointed away from”) the objects. Given the findings of previous research (Bayliss et al., 2006) we do not expect arrow cues to increase liking but rather expect the liking effect to be restricted to either gaze cues (the LOOK – IMAGE and LOOK – WORD groups). To test for the overall effect of presenting a directional cue, a further control condition (WORD – ONLY) was included in which any mention of the cue was removed and participants were presented with either the word “toward” or “away.” Finally, in Experiment 2 we tested for possible demand compliance.

## Experiment 1

### Materials and Methods

#### Participants

Participants were 205 students (125 female, 80 male) from the University of British Columbia. The mean age (and standard deviation) of the male and female participants in each experimental group are displayed in Table 1. The experiment was carried out in accordance with the ethical standards of the American Psychological Association and with the 1964 Helsinki declaration and its later amendments. The study was approved by the Psychology Ethics Committee at University of British Columbia, Vancouver. Written, informed consent was obtained from all participants in the study.

**Table 1:** The number (*N)*, mean (*M)* and standard deviation (*SD)* of the age of male and female participants as a function of group (LOOK – IMAGE, LOOK – WORD, POINT, ARROW, and WORD ONLY).

	Sex					
	Male			Female		
	*M*	*SD*	*N*	*M*	*SD*	*N*
LOOK – IMAGE	20.91	1.88	23	21.25	4.62	20
LOOK – WORD	19.50	1.65	22	20.32	1.84	22
POINT	21.93	1.07	14	22.83	3.27	23
ARROW	22.21	2.08	14	21.73	1.8	30
WORD – ONLY	22.43	2.44	7	22.33	2.77	30


#### Stimuli and Procedure

For the first LOOK – IMAGE group, the stimuli consisted of the images of 16 objects typically found in a garage (e.g., pliers and rake) and 16 objects typically found in a kitchen (e.g., electric whisk and saucepan). For the remaining groups (LOOK – WORD, POINT, ARROW, and WORD ONLY) words replaced the images used in the LOOK – IMAGE group. The images of objects were selected by the first author on the basis that they were easily recognizable as objects that might typically be found in either the garage or kitchen. For all subsequent conditions (LOOK – WORD, POINT, ARROW, and WORD – ONLY), the images were replaced with verbal descriptions of the objects (e.g., “Pliers”). All objects or verbal descriptions of objects were rated on a single sheet of paper (see Supplementary Material for the sheets used for the LOOK – IMAGE and LOOK – WORD groups). On one side of the paper a person was described as looking toward the object (e.g., “Michael looked toward the”). On the other side the person was described as looking away from the same object (e.g., “Michael looked away from the”). The text appeared above the object name. On each side, object names appeared in a fixed location in one of four columns. Each column contained one specific type of object. From left to right, the columns contained descriptions garage objects followed by 2 columns of kitchen objects and finally, the remaining 8 garage objects.

#### Procedure

Participants were randomly allocated to one of five groups (LOOK – IMAGE, LOOK –WORD, POINT, ARROW, and WORD ONLY). Testing took place in large classroom in groups of approximately 20–30 participants. The order in which a specific side of the sheet of paper was completed was counterbalanced across participants. For example, half of the participants in the LOOK group received the description of a person looking toward a specific object first, followed by a description of the same person looking away from the same objects (on the reverse of the sheet) whereas the remaining participants received the description in the reverse order. Participants were asked to rate the degree to which they liked the objects using the following 9-point scale: 1, do not like at all; 2, 3, 4, 5, 6, 7, 8, 9, like very much.

Participants were informed that there were no correct or incorrect answers. The scale appeared beneath each object name. In the POINT condition, the word “looked” was replaced by the word “pointed.” In the ARROW group the words “an arrow” replaced the names given to the actors and the word “pointed” was also used (e.g., “an arrow pointed toward the”). Finally, in the WORD ONLY condition, the description of the person or arrow looking or pointing was removed – participants received either the word “toward” or “away” above the object name.

### Results and Discussion

The mean liking ratings and standard deviations for each combination of cue direction and group are shown in Table 2. The mean liking ratings were analyzed in a cue direction (toward, away) X group (LOOK – IMAGE, LOOK – WORD, POINT, ARROW, and WORD ONLY) mixed ANOVA with cue direction as the within subjects variable. There was a main effect of cue direction, *F*(1,203) = 47.60, *p* < 0.0001 and a cue direction X group interaction, *F*(4,203) = 4.74, *p* < 0.005. The main effect of group was not significant, *F*(4,203) = 0.55, *p* = 0.70. Our prediction was that the cueing effect would be restricted to descriptions of eye gaze behavior (the LOOK groups) and therefore, we analyzed the interaction by testing for the simple main effect of cue direction (toward vs. away) for each group separately. Mean liking ratings for objects were higher in the toward condition compared to the away condition for the LOOK – IMAGE, *F*(1,43) = 15.32, *p* < 0.0001, LOOK – WORD, *F*(1,43) = 19.05, *p* < 0.0001, POINT, *F*(1,38) = 14.05, *p* < 0.0001 and ARROW group, *F*(1,43) = 5.95, *p* < 0.05 but not the WORD ONLY group, *F*(1,36) = 0.33, *p* = 0.57.

**Table 2:** Mean liking ratings as a function of group (LOOK – IMAGE, LOOK – WORD, POINT, ARROW, and WORD ONLY) and cue direction (toward and away).

		Toward		Away	
Experiment	*Example text*	*M*	*SD*	*M*	*SD*
LOOK – IMAGE^∗^	“Michael looked away...”	5.53	0.87	4.76	0.99
LOOK – WORD	“Michael looked away...”	5.60	0.92	4.70	1.08
POINT	“Michael pointed away...”	5.47	0.98	5.03	1.11
ARROW	“The arrow pointed away...”	5.45	0.91	5.17	0.91
WORD ONLY	“Toward” or “Away”	5.38	0.97	5.33	0.93


*^∗^In the LOOK – IMAGE experiment participants rated pictures of objects. All other groups read descriptions of objects.*

**FIGURE 1:**
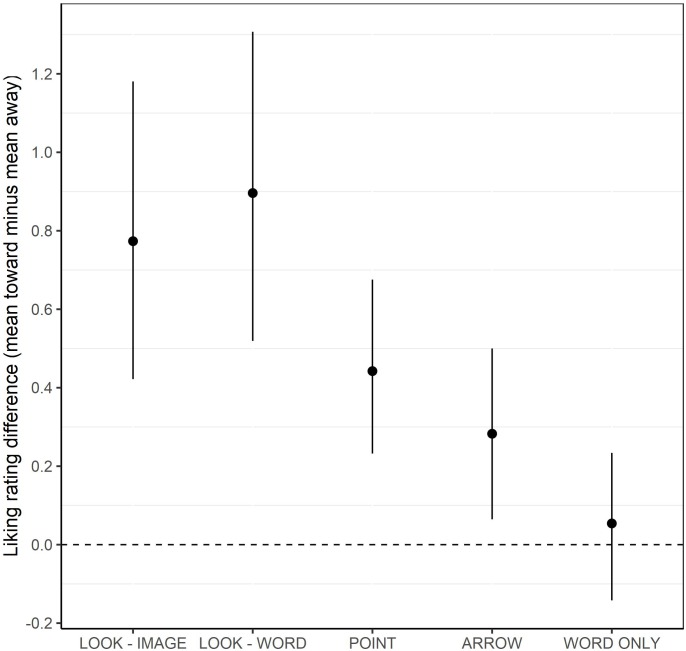
The mean cueing effect (mean toward liking rating minus mean away liking rating) with bootstrapped error bars (95% CI) as a function of cue type (LOOK – IMAGE, LOOK – WORD, POINT, ARROW, and WORD ONLY).

In addition, as can be seen in Figure 1, there were relative differences in the magnitude of the cue liking effect (mean liking ratings in the toward condition minus mean liking ratings in the away condition) as a function of group. Specifically, *post hoc* analyses of the cue liking effect (mean liking ratings toward minus mean liking ratings away) using the Tukey-Kramer adjustment for multiple pairwise comparisons showed that the magnitude of the liking effect was higher in the LOOK – IMAGE condition compared to the WORD ONLY group [difference = 0.71; 95% CI_adjusted_ (0.09–1.34), *p*_adjusted_ = 0.015] and also, higher in the LOOK – WORD group compared to both the ARROW [difference = 0.61; 95% CI_adjusted_ (0.01–1.21), *p*_adjusted_ = 0.041] and WORD ONLY [difference = 0.84; 95% CI_adjusted_ (0.21–1.46), *p*_adjusted_ = 0.002] groups.

## Experiment 2

We have shown that a variety of cues affect object liking, and moreover, the effect occurs for verbal descriptions of both the cue and the target object. Our interpretation is that individuals base their preference on previous experience – they know that people often look toward objects they like, and look away from objects they dislike, and people use this knowledge to help decide whether they like or dislike an object. An alternative interpretation is that the apparent effects are due to demand compliance – participants reported higher ratings for cued objects simply because they thought they were supposed to. The lack of an effect in the WORD ONLY group seems to rule out this possibility. However, it is possible that participants in this condition may not have had sufficient information to understand the implications of the word in isolation and consequently failed to rate the objects as liked more in the toward condition vs. the away condition.

To address the issue of demand compliance Bayliss et al. (2006) asked participants (during a post-experimental debrief) what they felt had influenced their ratings. In that study, participants never mentioned the cue, and appropriately the investigators concluded that participants did not work out the purpose of the study. Therefore, we attempted to replicate the LOOK – IMAGE in a new sample of participants who were asked (at debriefing) to list any factors that they thought may have may have influenced their ratings of items. The stimuli were identical to those used in the LOOK – IMAGE condition and also, the order in which a specific side of the sheet of paper was completed was counterbalanced across participants.

### Materials and Methods

#### Participants

There 74 participants (mean age = 20.43; *SD* = 4.04; 61 females and 13 males). The experiment was carried out in accordance with the ethical standards of the American Psychological Association and with the 1964 Helsinki declaration and its later amendments. The study was approved by the Psychology Ethics Committee at University of British Columbia, Vancouver. Written, informed consent was obtained from all participants in the study.

#### Design and Procedure

The design and procedure were identical to the LOOK – IMAGE condition in Experiment 1 except that participants were asked (at debriefing) to list any factors that they thought may have may have influenced their ratings of items.

### Results and Discussion

Following Bayliss et al. (2006) none of the participants mentioned direction of cue as having influenced their ratings but instead, reported basing their ratings on various aspects (e.g., usefulness) of the target objects. A paired samples *t*-test, showed that participants gave higher mean liking ratings in the toward condition (*M* = 4.90; *SD* = 1.07) compared to the away condition (*M* = 4.32; *SD* = 0.99), *t*(73) = 4.43, *p* < 0.001 [difference = 0.58; 95% CI (0.32–0.84)].

### General Discussion

The results show that increased liking for gazed-at objects is also found for verbal descriptions of gaze direction. Specifically, participants rated both images and verbal descriptions of objects as more likeable after reading a written description of a person looking toward rather than away from the objects. Although the results showed that the cue direction effect was not specific to eye gaze – the gaze liking effect was recorded for descriptions of pointing and also, for an arrow cue – the effect was largest in magnitude for descriptions of gaze behavior. In other words, the results offer partial support for the gaze specificity hypothesis because descriptions of individuals looking at objects produced the largest effect – eye gaze may not be unique but it is a highly effective cue.

The finding that non-social cues also exert influence contrasts with the lack of an effect for arrows reported previously in a study using an implicit measure of influence (Bayliss et al., 2006). As noted earlier this may because the original gaze-liking effect is much smaller than previously thought, and consequently, detecting such effect for arrow cues will be difficult unless the sample size is very large (*N* > 500; see Tipples and Pecchinenda, 2018). In contrast, in the current research, the effect was replicable and therefore, the current task is more suitable for testing for differences in magnitude across different cue types. Indeed, as already noted, the current data go some way to supporting the existence of a mechanism that is more sensitive to social vs. non-social cue differences because the effect of gaze on liking (in the LOOK WORD condition) was relatively greater than the arrow cue condition. This increased sensitivity for gaze cues may reflect a specifically social process that evolved to respond to gaze cues but has been co-opted to respond to other cue types.

In keeping with research designed to investigate mental state attribution, the effect of gaze on liking generalized to verbal stimuli, i.e., the effects do not depend on actual visual observation of either the cue or object stimuli. One interpretation of this finding is that the effects of cue stimuli on liking reflect existing knowledge. When forced to make a liking decision based on limited information participants rely on what they know about the relationship between cue direction and the likeability of objects. What knowledge is relevant to direction cues and object evaluation? Individuals may rely on knowledge of human motivation to either approach a rewarding object or avoid an unpleasant object when they read descriptions of individuals looking toward or away from an object. Put differently, participants may know that gazing-away from an object typically means that the object is not rewarding and therefore, they give the object a lower rating. A reliance on existing knowledge does not necessarily mean that the gaze-induced-liking is not rooted in a biologically based process. The tendency to use gaze and facial expressions as a basis for evaluating objects may still have a biological basis that is present in the early stages of human development.

Comparison of the WORD ONLY condition with the words “TOWARD” and “AWAY” with “LOOK – WORD” condition indicates that simply presenting motivational relevant words is not sufficient to record a gaze liking effect. Instead, the current findings suggest that sentence frame in which an actor is described as acting toward an object is needed to record the gaze-liking effect. One interpretation of this finding is that the gaze-liking effect requires propositional knowledge – a statement about the world and more specifically a statement about relationships in the world (“Michael looked *toward*...”). This finding accords with a growing recognition that propositional knowledge contributes to implicit evaluation processes (De Houwer, 2014). Specifically, according to the propositional model of implicit evaluation (De Houwer, 2014) automatic evaluation depends on the formation or activation of propositional knowledge. The current results are consistent with such an account.

Were our cue effects due to demand compliance? Put differently, did participants work out the purpose of the study and then respond in the manner expected by the experimenter? Following the methodology of Bayliss et al. (2006), our participants in Experiment 2 did not report using the cue to make their likeability judgments. Bayliss et al. (2006) concluded that the lack of self-reported knowledge indicated that their participants had not worked out the purpose of the study. Against this high standard we also conclude that participants in our investigation had not worked out the purpose of the study, that is, the effects were not due to demand compliance.

In conclusion we have shown that shifting attention to an object is not necessary for eye gaze cues to influence object liking – the effect occurs for verbal descriptions of eye gaze. Furthermore, a variety of cues can influence object liking – the effect is not restricted to eye gaze cues – and moreover, the effects were not due to demand compliance. In sum, our results suggest a separate, conceptually based route, by which cue direction can affect object evaluation. Whether this route is also responsible for previous findings of liking that employed the gaze cue paradigm is an exciting issue for future investigation. One thing is very clear, while the conceptually based route may explain past findings of liking with the gaze cue paradigm, those past findings with the gaze cue paradigm are unable to account for the present data insofar as they are grounded on the assumptions that an external gaze cue and a shift of visuo-spatial attention toward or away from an object are necessary to trigger a liking effect.

## Data Availability

The datasets generated for this study are available on request to the corresponding author.

## Author Contributions

JT and AK discussed the design of the experiments. MD arranged for data collection. JG collected the data. JT analyzed the data and wrote the manuscript. AK and MD commented on various drafts.

## Conflict of Interest Statement

The authors declare that the research was conducted in the absence of any commercial or financial relationships that could be construed as a potential conflict of interest.
