# Supplementary material for: Verbal Descriptions of Cue Direction Affect Object Desirability
Source: Front Psychol. 2019 Mar 11;10:471. doi: 10.3389/fpsyg.2019.00471 (PMC6421290; doi:10.3389/fpsyg.2019.00471)
Supplement: Supplementary file 1 [file Image_1.pdf]

A. Please read the text above the pictures and then rate how much you like the objects by circling a number between 1 and 9 USE following scale: 1=Do not like at all, 2, 3, 4, 5, 6, 7, 8, 9=like very much. The text describes whether participants are looking TOWARD or AWAY from the objects. There are no correct/incorrect answers.

First please circle your sex MALE FEMALE and write your age_ _ _ (years) SUB NO:

Michael looked TOWARD the<br>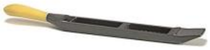<br>1 2 3 4 5 6 7 8 9	Judy looked AWAY from the<br>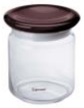<br>1 2 3 4 5 6 7 8 9	Megan looked AWAY from the<br>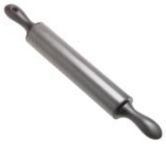<br>1 2 3 4 5 6 7 8 9	Justin looked TOWARD the<br>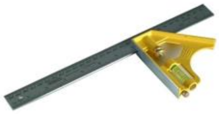<br>1 2 3 4 5 6 7 8 9
Jennifer looked TOWARD the<br>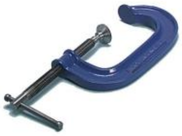<br>1 2 3 4 5 6 7 8 9	Simon looked AWAY from the<br>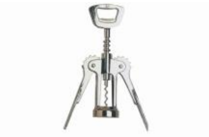<br>1 2 3 4 5 6 7 8 9	Robert looked AWAY from the<br>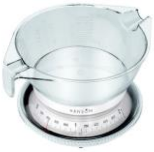<br>1 2 3 4 5 6 7 8 9	Melissa looked TOWARD the<br>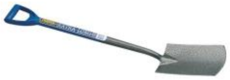<br>1 2 3 4 5 6 7 8 9
Matthew looked TOWARD the<br>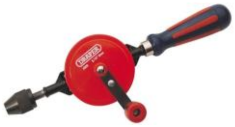<br>1 2 3 4 5 6 7 8 9	Sarah looked AWAY from the<br>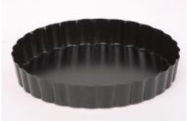<br>1 2 3 4 5 6 7 8 9	Lucy looked AWAY from the<br>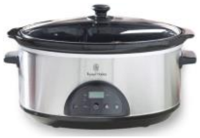<br>1 2 3 4 5 6 7 8 9	John looked TOWARD the<br>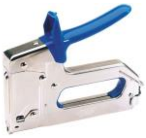<br>1 2 3 4 5 6 7 8 9
Stephanie looked TOWARD the<br>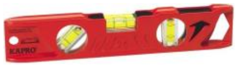<br>1 2 3 4 5 6 7 8 9	Joshua looked AWAY from the<br>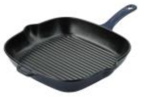<br>1 2 3 4 5 6 7 8 9	Joseph looked AWAY from the<br>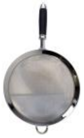<br>1 2 3 4 5 6 7 8 9	Lauren looked TOWARD the<br>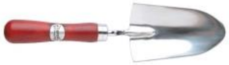<br>1 2 3 4 5 6 7 8 9
David looked TOWARD the<br>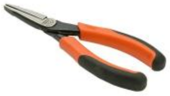<br>1 2 3 4 5 6 7 8 9	Nicole looked AWAY from the<br>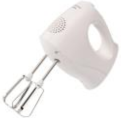<br>1 2 3 4 5 6 7 8 9	Rachel looked AWAY from the<br>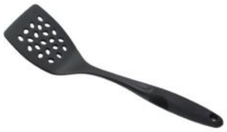<br>1 2 3 4 5 6 7 8 9	Ryan looked TOWARD the<br>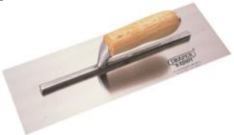<br>1 2 3 4 5 6 7 8 9
Heather looked TOWARD the<br>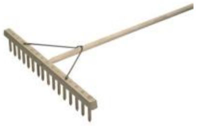<br>1 2 3 4 5 6 7 8 9	Andrew looked AWAY from the<br>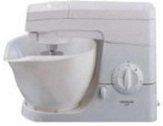<br>1 2 3 4 5 6 7 8 9	Brian looked AWAY from the<br>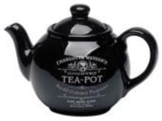<br>1 2 3 4 5 6 7 8 9	Tiffany looked TOWARD the<br>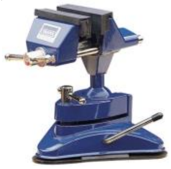<br>1 2 3 4 5 6 7 8 9
Daniel looked TOWARD the<br>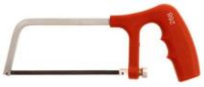<br>1 2 3 4 5 6 7 8 9	Elizabeth looked AWAY from the<br>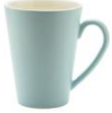<br>1 2 3 4 5 6 7 8 9	Emily looked AWAY from the<br>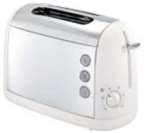<br>1 2 3 4 5 6 7 8 9	William looked TOWARD the<br>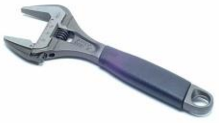<br>1 2 3 4 5 6 7 8 9
Samantha looked TOWARD the<br>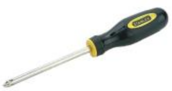<br>1 2 3 4 5 6 7 8 9	James looked AWAY from the<br>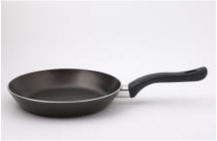<br>1 2 3 4 5 6 7 8 9	David looked AWAY from the<br>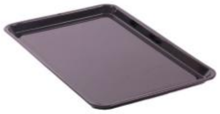<br>1 2 3 4 5 6 7 8 9	Kimberly looked TOWARD the<br>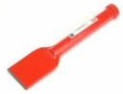<br>1 2 3 4 5 6 7 8 9

B. Please read the text above the pictures and then rate how much you like the objects by circling a number between 1 and 9 USE following scale: 1=Do not like at all, 2, 3, 4, 5, 6, 7, 8, 9=like very much. The text describes whether participants are looking TOWARD or AWAY from the objects. There are no correct/incorrect answers.

First please circle your sex MALE FEMALE and write your age_ _ _ (years)

SUB NO:

Michael looked AWAY from the<br>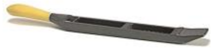<br>1 2 3 4 5 6 7 8 9	Judy looked TOWARD the<br>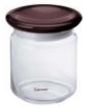<br>1 2 3 4 5 6 7 8 9	Megan looked TOWARD the<br>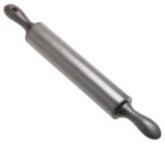<br>1 2 3 4 5 6 7 8 9	Justin looked AWAY from the<br>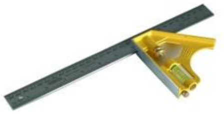<br>1 2 3 4 5 6 7 8 9
Jennifer looked AWAY from the<br>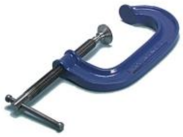<br>1 2 3 4 5 6 7 8 9	Simon looked TOWARD the<br>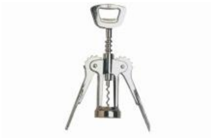<br>1 2 3 4 5 6 7 8 9	Robert looked TOWARD the<br>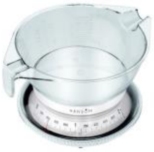<br>1 2 3 4 5 6 7 8 9	Melissa looked AWAY from the<br>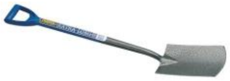<br>1 2 3 4 5 6 7 8 9
Matthew looked AWAY from the<br>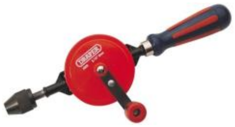<br>1 2 3 4 5 6 7 8 9	Sarah looked TOWARD the<br>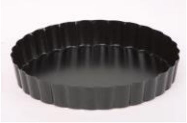<br>1 2 3 4 5 6 7 8 9	Lucy looked TOWARD the<br>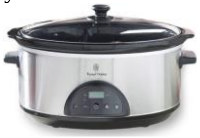<br>1 2 3 4 5 6 7 8 9	John looked AWAY from the<br>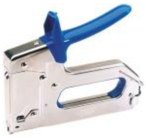<br>1 2 3 4 5 6 7 8 9
Stephanie looked AWAY from the<br>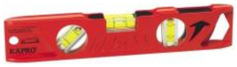<br>1 2 3 4 5 6 7 8 9	Joshua looked TOWARD the<br>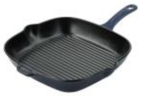<br>1 2 3 4 5 6 7 8 9	Joseph looked TOWARD the<br>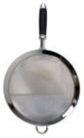<br>1 2 3 4 5 6 7 8 9	Lauren looked AWAY from the<br>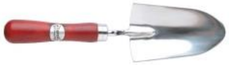<br>1 2 3 4 5 6 7 8 9
David looked AWAY from the<br>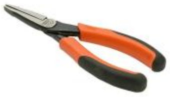<br>1 2 3 4 5 6 7 8 9	Nicole looked TOWARD the<br>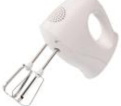<br>1 2 3 4 5 6 7 8 9	Rachel looked TOWARD the<br>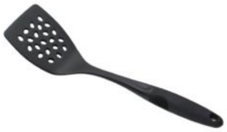<br>1 2 3 4 5 6 7 8 9	Ryan looked AWAY from the<br>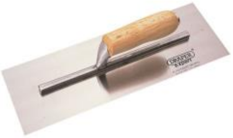<br>1 2 3 4 5 6 7 8 9
Heather looked AWAY from the<br>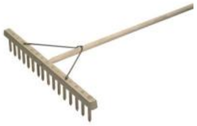<br>1 2 3 4 5 6 7 8 9	Andrew looked TOWARD the<br>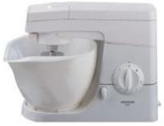<br>1 2 3 4 5 6 7 8 9	Brian looked TOWARD the<br>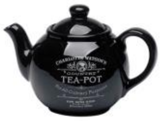<br>1 2 3 4 5 6 7 8 9	Tiffany looked AWAY from the<br>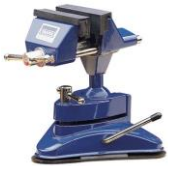<br>1 2 3 4 5 6 7 8 9
Daniel looked AWAY from the<br>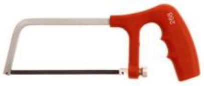<br>1 2 3 4 5 6 7 8 9	Elizabeth looked TOWARD the<br>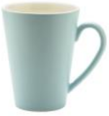<br>1 2 3 4 5 6 7 8 9	Emily looked TOWARD the<br>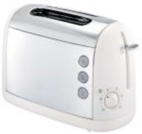<br>1 2 3 4 5 6 7 8 9	William looked AWAY from the<br>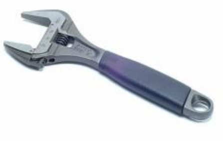<br>1 2 3 4 5 6 7 8 9
Samantha looked AWAY from the<br>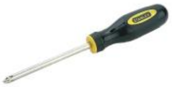<br>1 2 3 4 5 6 7 8 9	James looked TOWARD the<br>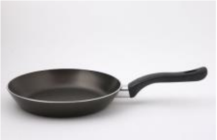<br>1 2 3 4 5 6 7 8 9	David looked TOWARD the<br>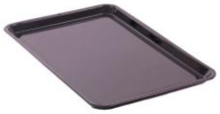<br>1 2 3 4 5 6 7 8 9	Kimberly looked AWAY from the<br>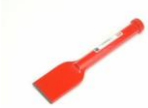<br>1 2 3 4 5 6 7 8 9
